# Pesticides in fine airborne particles: from a green analysis method to atmospheric characterization and risk assessment

**DOI:** 10.1038/s41598-017-02518-1

**Published:** 2017-05-23

**Authors:** Madson M. Nascimento, Gisele O. da Rocha, Jailson B. de Andrade

**Affiliations:** 1 Instituto de Química, Universidade Federal da Bahia, Campus de Ondina, 40170-115 Salvador-BA, Brazil; 2 Instituto Nacional de Ciência e Tecnologia em Energia e Ambiente - INCT, Universidade Federal da Bahia, 40170-115 Salvador, BA, Brazil; 3 Centro Interdisciplinar em Energia e Ambiente - CIEnAm, Universidade Federal da Bahia, 40170-115 Salvador, BA, Brazil

## Abstract

The intensive use of pesticides such as herbicides, insecticides, fungicides and acaricides has been lead to ubiquitous contamination, being present not only in soils, water bodies and/or crops, but also in the atmosphere. Considering the massive amount of pesticides employed globally, together to their persistence, this may be an important concern regarding air quality and human health worldwide. In the present study we developed a green sensitive sample preparation method for determination of nine organophosphates, two pyrethroids, one carbamate, and one strobirulin in PM2.5 collected in a tropical coastal area in the Southern Hemisphere for the first time. Extraction of PM2.5 sample masses, as low as 206 µg, were performed in a miniaturized device using 500 μL of a mixture containing 18% acetonitrile in dichloromethane followed by sonication for 23 minutes and injection into GC-MS. A total of 12 pesticides were identified and quantified successfully, among them, eight banned pesticides. A risk assessment exposure and cancer risk for possible carcinogenic pesticides (bifenthrin, malathion, parathion and permethrin) were performed for exposure of adults, children and infants. Hazard Quotient and cumulative exposure for organophosphate and pyrethroid pesticides were less than 1, showing that cumulative risk is within acceptable range.

## Introduction

Atmospheric particles with aerodynamic diameter lower than 2.5 µm (PM2.5) are the most hazardous for human health because they are directly linked to respiratory diseases due their high capacity to penetrate deep in the lungs^1–3^. According to Lelieveld *et al*.^1^, premature deaths from inhalation of PM2.5 in outdoor environments were 3.3 million people worldwide in 2010 and it is estimated to be more than 6.5 million premature deaths by 2050.

Particulate matter (PM) is a complex mixture composed by solid/liquid organic and inorganic substances. PM generally has atmospheric lifetimes ranging from 3 to 10 days, in typical conditions. Within this lifetime, particles can be transported over several thousands of kilometers, depending on the meteorological conditions, even being possible to reach remote regions^4^. In this way, if pesticides or other hazardous substances are adsorbed on the PM, they may also be transported together through wind over long distances, becoming hazardous materials through inhalation to populations located far away from the sources^5, 6^. Considering the amount of pesticides employed globally (estimated to be 1 to 2.5 million tons of active pesticides ingredients)^6, 7^, together to their persistence in the environment and bioaccumulative characteristics^8^, this may be an important concern regarding air quality and human health worldwide.

Pesticides are bioactive compounds widely used for control, prevention or elimination of plagues (from either animal, plant or microbiological origin), which could potentially cause damage to food production and agricultural commodities^9^. The intensive use of pesticides such as herbicides, insecticides, fungicides and acaricides has been lead to ubiquitous contamination, being able not only to be present in soils, water bodies and/or crops, but also in the atmosphere^10^. The consumption of pesticides has been increased in the last years, mainly in agricultural sector, where high productivity is an exigency for market supply^11^. However, the increase in the pesticide consumption became an alarming case of public health. Because of their toxicities and prolonged exposition (either voluntarily or not) of humans to these compounds, this is not only directly linked to a higher cancer incidence^12^, but also is suspected to cause disruption of the endocrine system^7^, and to affect the central nervous system, being a risk for Alzheimer’s and Parkinson’s diseases^13–15^. Indeed, pesticides are suspected to cause a large spectrum of different other diseases^16, 17^. Children are more susceptible to contamination by pesticides than adults, once their physiological system still is in development^14, 18^. Currently used pesticides (CUPs) are actually utilized pesticides including also those that were banned but have potentially been used illegally in some countries^14, 19, 20^. Some pesticides such as carbofuran, demeton-O, diazinon, disulfoton, ethion, fenthion, parathion, permethrin and sulfotep are currently prohibited by the European Union (EU) but many of them are still used, especially in Brazil.

Pesticides are widely and massively used in Brazil, which is the world leader of pesticide consumption since 2012^21, 22^. For instance, in 2014, 508,557 tonnes of pesticides in terms of active ingredients in Brazil^23^. Most of it was probably used in about 568 thousand km^2^ of cultivated area (2014)^24^ and about 167 thousand km^2^ for breeding cattle (2014/2015)^25^. Yet, in 2016 800,000 L of malathion, 250,000 kg pyriproxyfen, 8,196 L lambda cyalotrin, 26,412 kg etofenprox, and 20,000 L bendiocarb were purchased by the Health Ministry for combating the outbreak of *Aedes aegypti*, which is the transmitter of many diseases, such as dengue, yellow fever, zika and chikungunya^26^. However, there is still limited information regarding the occurrence of pesticides associated to fine particles in Brazil or around the world.

During application of pesticides, a significant portion of the applied dosage does not reach the target, being transported to others areas through spray drift^8, 27^. Depending on some of their physicochemical properties, such as vapor pressure, these pesticides may easily attain into atmosphere and be distributed between gaseous phase and/or particles. Volatile pesticides may reach the atmosphere soon after their application or by volatilization from plants and soils. In turn, semi-volatile pesticides may be simultaneously present in both particulate and gaseous phases. It is known that compounds with vapor pressure higher to 10^−2^ Pa are principally observed in the gas phase and those with vapor pressure lower than 10^−5^ Pa are almost exclusively found in the atmospheric particulate phase^8, 10, 28, 29^.

Inhalation is the most disturbing means of exposure to pesticides and exposure risk assessment studies are required. In recent studies Yusà *et al*.^30^ and Lopéz *et al*.^31^ have evaluated the exposure of adults, children and infants to pesticides in particulate matter. However, these studies considered only particles within larger sizes (PM10), which is not directly linked to possible health endpoints in humans. Yet, limited information is available to pesticides in atmospheric fine aerosols. Nevertheless, pesticides are present in the air in very low concentrations (from pg m^−3^ to low µg m^−3^) and therefore, fast and efficient methods of extraction are necessary.

In spite the fact Soxhlet is the official and conventional extraction method for pesticides analysis in air samples^32^, the main disadvantages in using it are the long extraction time (6–24 h), the large volume of solvent consuming (250–700 mL)^10^, the use of a large sample size in order to meet the instrumental limit of detections (LOD), the difficulty of automation or miniaturization, thus resulting in large quantities of organic solvents waste disposal and low analytical frequency^33^.

Due to the current miniaturization trend of sample preparation systems and the adoption of the Green Chemistry principles, more environmental-friendly procedures have been utilized for pesticides extraction^34^. For instance, Pressurized Liquid Extraction (PLE)^35–39^ and Microwave Assisted Extraction (MAE)^19, 31, 40^ have been used currently for pesticides extraction in particulate matter since they offer low consumption of organic solvents with high efficiency. However, these methods are not simple and require expensive and sophisticated apparatus^34^. On the other hand, miniaturized systems based in the Ultrasonic-Assisted Extraction (UAE) have been considered a simple and inexpensive alternative for pollutant extraction from particulate matter^33, 41^. In the UAE, acoustic cavitation generated by the ultrasound energy produces peaks of temperature and pressure on a microscopic scale that can reach 5000 K and 50 MPa, respectively^42^. These extreme conditions increase the extraction yield, facilitating the solvent penetration in the sample matrix and then improving mass transfer, and, consequently, resulting more efficient extractions^43, 44^.

This study aimed to develop a simple and miniaturized ultrasound assisted-extraction method for determination of 13 multiclass pesticides (Supplementary Table 1) in the fine particulate matter (PM2.5). The procedure was carried out by ultrasound assisted-extraction of PM2.5 samples using a miniaturized extractor device followed by a direct injection into GC-MS system. After optimization of the extraction procedure, method validation was performed in order to meet satisfactory figures for the following parameters: linear range, linearity, limit of detection (LOD), limit of quantification (LOQ), precision, and accuracy. Finally, the present method was applied to determine the pesticides in real PM2.5 samples collected in three sites located in the Todos os Santos Bay, Northeastern Brazil. Finally, we also evaluated the risk exposure for the studied pesticides by calculating the daily inhalation exposure (DIE), the hazard quotient (HQ), the possible cumulative effect of a group of pesticides by the hazard index (HI) and the risk of cancer for infants, children and adults. We have critically discussed our results in face of the current knowledge about pesticides in airborne particles.

## Results and Discussion

### Comparison of two different extraction devices

In order to evaluate the influence of two different types of microextraction devices, an extraction of blank samples (500 μL ACN/DCM) was carried out using two different microextractors: microextraction device with a polypropylene chamber (Whatman Mini-UniPrep plastic version device) consisting of both the plunger and the chamber being polypropylene-made and micro-extraction device with borosilicate glass chamber (Whatman Mini-UniPrep G2) with a polypropylene plunger and a borosilicate glass chamber. After this test we verified that the microextraction device (Whatman Mini-UniPrep G2) resulted in better results, with less interfering compounds as shown in the chromatogram in Supplementary Figure 1. Since the extraction solvent mixture is 18% ACN and 82% DCM, during the extraction step these solvents probably leach monomers from the device with polypropylene chamber. In this way, in order to avoid the extraction of these interferents, hereafter we only employed the borosilicate glass chamber microextractor (Whatman Mini-UniPrep G2) in our analyses. A scheme of the extraction procedure is shown in Supplementary Figure 2.

### Chromatographic analysis

**Figure 1:**
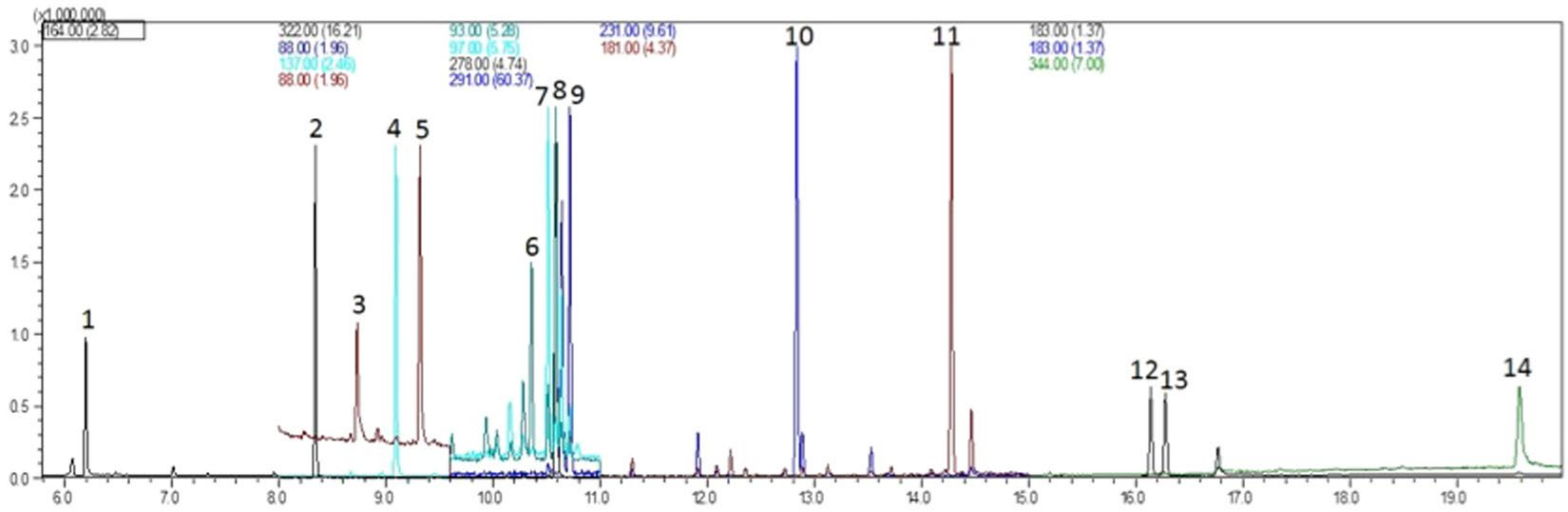
Chromatogram of a 7.5 µg L^−1^ standard solution containing 14 compounds analyzed by GC-MS in SIM mode. Compounds listed in elution order: [1] Carbofuran (6.20 min), [2] sulfotep (8.34 min), [3] demeton-O (8.73 min), [4] diazinon (9.08), [5] disulfoton (9.32 min), [6] malathion (10.36 min), [7] chloropyrifos (10.52 min), [8] fenthion (10.59 min), [9] parathion (10.64 min), [10] ethion (12.84 min), [11] bifenthrin (14.29 min), [12] permethtin I (16.15 min), [13] permethrin II (16.29 min), [14] azoxystrobin (19.58 min).

The optimization of pesticides separation was performed in full scan mode and all compounds were separated with satisfactory resolution within 20 min of total runtime (Fig. 1). In order to improve compounds detection in the SIM mode, three more intense ions of each compound were chosen through the mass spectrum obtained in the Full Scan mode (Table 1). However, in the case of malathion and chloropyrifos, it was necessary to replace the base ions (m/z 93 and 97, respectively), due to the presence of interfering compounds eluting at the same retention time. Instead, qualifier ions with m/z 173 and 197 were used for these compounds, respectively. In this way, after carefully choosing specific m/z ions, we could better approach unequivocal identification for them (Fig. 1).

**Table 1:** Parameters of GC-MS analysis in SIM mode for the selected pesticides.

Retention time window (min)	Pesticide	TR (min)	Target ion^a^ (m/z)	Qualifier ion #1^b^ (m/z)	Qualifier ion # 2^b^ (m/z)
5.70–8.00	Carbofuran	6.20	164	149	131
8.00–9.60	Sulfotep	8.34	322	97	65
	Demeton-o	8.73	88	60	—
	Diazinon	9.08	137	179	304
	Disulfoton	9.32	88	60	97
9.60–11.00	Malathion	10.36	173	93	125
	Chloropyrifos	10.52	197	97	125
	Fenthion	10.59	278	125	109
	Parathion	10.64	291	109	97
11.00–15.00	Ethion	12.84	231	97	384
	Bifenthrin	14.29	181	165	166
15.00–20.00	Permethrin I	16.15	183	163	165
	Permethrin II	16.29	183	163	165
	Azoxystrobin	19.58	344	388	372

^a^ion base, used for quantification purposes; ^b^reference ions, used for improving pesticide identification.

### Extraction procedure and matrix effect study as an approach to accuracy

In order to evaluate the extraction efficiency using the miniaturized extraction device, recovery tests were performed through spikes of blank filters. Initially, recovery values higher than 140% were observed for fenthion (149%), bifenthrin (184%), permethrin I (186%), azoxystrobin (198%), ethion (201%) and parahion (265%) showing signs of matrix enhancement (signal enhancement). Thus, a matrix study was performed as described by Matuszewski, Constanzer e Chavez-Eng^45^. Two different sets of pesticide standard solutions (n = 5) were prepared (set A: standards solution prepared in the extraction solvent; B: standards solution prepared in the extract of the blank filters). The presence or absence of the matrix effect (ME) was evaluated considering the relation of the peak areas of the patterns in the two sets of solutions (ME% = B/A × 100). When ME > 100% there is matrix-induced signal enhancement and when ME < 100% there is signal suppression^45^. Most of the pesticides presented a signal enhancement with ME% values ranging from 104% (carbofuran) to 379% (demeton-O). For chloropyrifos (92.9%) and sulfotep (98.6%) a small suppressive effect was observed. The matrix-induced signal enhancement is common in pesticide analyzes by GC-MS and occurs due to competition between matrix constituents and analytes by the active sites in the sample introduction systems^46^. In order to minimize matrix interference, matrix-matching calibration was used. Then, the calibration curves were constructed by diluting the standard solutions in the blank filter extracts. As shown in Table 2, this strategy led to a reduction of the matrix effect, where the recoveries for all compounds did not exceed 124% with relative standard deviation ≤20% for all pesticides. With the exception of ethion, which presented a low recovery percentage at lower and intermediate levels (31.2% and 63.0%, respectively), recoveries ranged from 70.2% (fenthion) to 124% (chloropyrifos), with RSD ≤ 20% for all pesticides at the three concentration levels were obtained. We also tried recovery tests by adding a known amount of each pesticide standard to SRM 1649b. Although this reference material is not certified to the analyzed pesticides in this study, we did this experiment in order to try our extraction procedure in recovering the studied pesticides from a real atmospheric particulate matter matrix. In this case, recovery levels ranged from 54.9% (chloropyrifos) to 140% (permethrin II). These results also are considered satisfactory within the experimental error.

**Table 2:** Mean recovery of pesticides in blank filters (n = 2) and NIST Standard Reference Material urban dust 1649b.

Compound	Level 1 (1.5 µg L^−1^)		Level 2 (3.5 µg L^−1^)		Level 3 (6.5 µg L^−1^)		Recovery test in urban dust SRM1649b		
	Recovery	RSD	Recovery	RSD	Recovery	RSD	Standard addition	Found values	Recovery
	(%)	(%)	(%)	(%)	(%)	(%)	µg L^−1^	µg L^−1^	(%)
Azoxystrobin	73.6	5.2	72.9	6.9	111	12.1	18.0	18.8	104
Bifenthrin	70.6	8.4	85.5	3.3	101	8.6	18.0	18.4	102
Carbofuran	101	7.5	93.0	2.4	114	6.6	18.0	17.3	96.4
Chloropyrifos	86.6	2.0	124	8.9	92.3	1.6	18.0	9.9	54.9
Demeton-o	71.9	0.4	65.4	4.4	70.5	2.5	18.0	17.9	99.4
Diazinon	85.6	4.3	99.4	5.8	116	2.6	18.0	17.1	95.2
Disulfoton	66.7	2.6	81.1	6.7	66.7	2.6	18.0	14.6	80.8
Ethion	31.2	10.6	63.0	1.2	87.3	4.5	18.0	17.5	97.0
Fenthion	70.2	3.3	97.7	4.5	115	7.5	18.0	16.0	89.1
Malathion	70.2	5.5	89.0	0.4	92.3	1.6	18.0	24.1	134
Parathion	108	13.4	104	7.2	101	20.0	18.0	15.6	86.6
Permethrin I	111	2.6	106	8.0	111	7.2	18.0	18.9	105
Permethrin II	106	0.3	107	3.9	112	3.8	18.0	25.3	140
Sulfotep	71.9	0.4	98.2	2.2	104	19.1	18.0	16.5	91.7

For comparison purposes, the percentages of recovery using the proposed procedure were similar to those obtained by Coscollà *et al*.^29, 47, 48^ that determined CUPs using MAE (30 mL solvent, 20 min extraction) and obtained recovery values ranging from 48 to 120% (RSD < 30%) for PM10 samples, and 70.1 to 115.4% (RSD < 27%) and 73 to 116% (RSD < 20%), for PM2.5 samples. Compared with ultrasound assisted extraction techniques (UAE), Borrás *et al*.^49^ employed UAE (total of 20 mL solvent, 20 min extraction) to extract 16 pesticides of different chemical classes in the vapor and particulate phases. More recently, Beristain-Montiel *et al*.^41^ employed the UAE (total of 10 mL, 10 min) with microscale cell to extract POPs in PM2.5 samples. However, the use of miniaturized extraction devices in the present study provided significant advantages over the procedures cited such as: simplicity, minimum solvent consuming (500 μL), minimum sample size (average particle mass: 687 μg, ranging from 206 μg to 1564 μg), reduced extraction time (23 minutes), and high analytical speed. Since that extraction and filtration procedure is simultaneously performed on the miniaturized device by itself as well as the relation solvent-volume-to-sample-particle-mass employed during extraction result in extracts with no need for further clean up nor additional preconcentration steps. Additionally, after minimizing matrix-effect and reaching satisfactory recovery levels with both spiked blank filters and urban dust SRM 1649b, we may say this method is accurate.

### Method validation

The method validation parameters are presented in Table 3. Matrix-matching calibration curves showed good determination coefficients (R^2^) with values ranging from 0.963 to 0.998. It was observed a linear region for all calibration curves with no evidence of significance to lack of fit (p < 0.05) at 95% confidence level.

**Table 3:** Method validation parameters.

Compound	Linear range (µg L^−1^)	Linearity (R²)	Regression ( ± Standard Error)	*p-value* ^a^ (lack of fit test)	Intraday (n = 9)	Interday (n = 27)	LOD (pg m^−3^)	LOQ (pg m^−3^)	LOD (pg)	LOQ (pg)
Azoxystrobin	0.51–6.5	0.998	y = 5437 _(± 69)_x − 271 _(± 280)_	0.4506	10.7	12.9	2.2	7.5	0.15	0.51
Bifenthrin	0.98–7.5	0.997	y = 26943 ( ± _608)_x − 9093 _(± 2642)_	0.4132	13.4	11.1	4.2	14.3	0.29	0.98
Carbofuran	0.83–7.5	0.997	y = 4951 _(± 102)_x − 1085 _(± 445)_	0.0984	4.76	16.7	3.6	12.2	0.25	0.83
Chloropyrifos	1.23–7.5	0.991	y = 3003 _(± 80)_x − 619 _(± 369)_	0.2261	7.31	13.6	3.7	12.4	0.37	1.23
Demeton-o	0.86–6.5	0.994	y = 15895 _(± 341)_x + 509 _(± 1374)_	0.1297	10.5	9.25	3.8	12.6	0.26	0.86
Diazinon	0.99–7.5	0.997	y = 14087 _(± 381)_x − 4353 _(± 1395)_	0.2377	8.91	12.7	4.3	14.5	0.30	0.99
Disulfoton	0.55–7.5	0.998	y = 18835 _(± 225)_x − 617 _(± 1040)_	0.1478	2.23	9.61	2.4	8.1	0.17	0.55
Ethion	0.61–7.5	0.997	y = 6934 _(± 98)_x + 717 _(± 426)_	0.2251	8.36	8.85	2.7	9.0	0.18	0.61
Fenthion	1.51–7.5	0.989	y = 12452 _(± 434)_x − 3530 _(± 1884)_	0.1767	14.0	11.4	6.6	22.1	0.45	1.51
Malathion	0.85–7.5	0.997	y = 2932 _(± 57)_x − 723 _(± 250)_	0.0734	8.78	11.9	3.6	12.2	0.26	0.85
Parathion	0.84–7.5	0.997	y = 756 _(± 14)_x + 95 _(± 64)_	0.7391	7.74	9.61	3.7	12.3	0.25	0.84
Permethrin I	3.64–6.5	0.972	y = 15512 _(± 1229)_x − 13445 _(± 5663)_	0.3492	15.3	15.9	15.9	53.3	1.09	3.64
Permethrin II	4.10–7.5	0.963	y = 14760 _(± 1305)_x − 12075 _(± 6012)_	0.0770	7.03	8.67	17.8	60.0	1.22	4.10
Sulfotep	0.71–7.5	0.997	y = 2196 _(± 36)_x − 148 _(± 156)_	0.4970	15.1	11.1	3.1	10.4	0.21	0.71

^a^p-value (lack of fit test) = p value (p < 0, 05) for lack of fit test.

LOD values ranged from 2.2 pg m^−3^ (azoxystrobin) to 17.8 pg m^−3^ (permethrin II), whereas LOQ ranged from 7.5 pg m^−3^ to 60 pg m^−3^ for these same pesticides, respectively. Since LOD and LOQ in terms of atmospheric levels (pg m^−3^) are dependent on sampling parameters (sampling time, flow rate and total volume of sampled air) we also calculated LOD and LOQ in terms of absolute mass units (pg). In this case, LOD and LOQ ranged from 0.15 to 1.22 pg and from 0.51 to 4.10 pg, for azoxystrobin and permethrin II, respectively. The LOD in this work was comparable to those reported by Borrás *et al*.^49^ who found values ranging from 50 to 380 pg. Regarding precision, relative standard deviation (RSD) values for intraday precision ranged from 2.23% (disulfoton) to 14.0% (fenthion) and from 8.67 (disulfoton) to 16.4% (carbofuran) for interday precision, showing the instrumental variations were acceptable for trace level analysis of complex matrix samples^50^.

### Application to real samples

**Figure 2:**
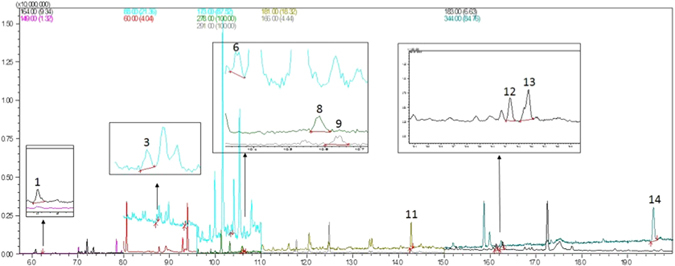
Chromatogram of a real PM2.5 sample collected in the Naval Base site. The following pesticides were detected: [1] Carbofuran (6.20 min), [3] demeton-O (8.73 min), [6] malathion (10.36 min), [8] fenthion (10.59 min), [9] parathion (10.64 min), [11] bifenthrin (14.29 min), [12] permethtin I (16.15 min), [13] permethrin II (16.29 min), [14] azoxystrobin (19.58 min).

The proposed procedure was applied in the determination of 13 pesticides in 41 PM2.5 samples collected at three different sites (Ilha de Maré, Naval Base and Ilha de Itaparica) in the Todos os Santos Bay region. A chromatogram of a real sample is shown in Fig. 2.

Considering all sampling sites, 12 of the 13 pesticides were detected with frequencies above 14% (Table 4). The average concentration of pesticides ranged from 20 to 315 pg m^−3^. Insecticides and acaricides such as carbofuran, malathion and permethrin were detected with higher frequencies (ranging from 70.7% to 100%). These pesticides are relatively volatile, with vapor pressure (Vp) ranging from 7.0 × 10^−3^ mPa (permethrin) to 3.1 mPa (malathion). Carbofuran and permethrin were often found in the particulate phase^19, 30, 38^, which justifies the high detection frequency in PM2.5 samples. However, it is noteworthy to mention, these pesticides are currently banned by the EU. On the other hand, malathion, which is permitted by the EU, is widely used in the Southern region of the State of Bahia, mainly against ants attacking cacao (*Theobroma Cacao L*.). But probably the employment of malathion for combating the zika, dengue, yellow fever and chikungunya outbreaks in Brazil may contribute to explain the even increasing levels of this pesticide in PM2.5 samples in a near future.

**Table 4:** Concentration of pesticides detected in all sampling sites (Maré Island, Itaparica Island and Naval Base).

Pesticide	Frequency of detection (%)^a^	Average^b^ (pg m^−3^)	Range (pg m^−3^)
Azoxystrob	31.7	59.2	9.71–184^b^
Bifenthrin	56.1	39.2	14.5–72.5^b^
Carbofuran	80.5	20.0	12.3–37.1^b^
Chloropyrifos	24.4	35.4	23.8–47.1^b^
Demeton-O	43.9	28.7	16.1–55.5^b^
Diazinon	34.1	—	nd − < LQ
Disulfoton	14.6	—	nd − < LQ
Ethion	63.4	35.3	9.20–94.1^b^
Fenthion	26.8	—	nd − < LOQ
Malathion	70.7	27.9	13.0–66.7^b^
Parathion	24.4	55.6	18.1–95.8^b^
Permethrin^c^	95.1	315	62.1–945^b^

^a^Frequency of detection was calculated considering samples with concentration above the limit of detection (LOD).

^b^Were considered only concentration above the limit of quantification (LOQ).

^c^Sum of the permethrin I and permethrin II isomers.

Ethion and bifenthrin were detected with frequencies above 50% with concentrations varying from 35.3 to 39.2 pg m^−3^, respectively. Bifenthrin (Vp = 1.78 × 10^−2^ mPa) is almost exclusively distributed in the particulate phase. In the State of Bahia, bifenthrin is used as an insecticide in fruit and vegetable crops in the mesoregion of São Francisco Valley. The pesticides azoxytrobin, demeton-O, diazinon, disulfoton, chloropyrifos, fenthion and parathion were found with frequencies ranging from 14.6 to 43.9%. Volatile pesticides such as demeton-O, chloropyrifos and parathion were found in concentrations greater than 16 pg m^−3^. On the other hand, diazinon, disulfoton and fenthion showed concentrations below the LOQ. Sulfotep was not detected in any PM2.5 samples and therefore is not shown in the Table 4. This can be attributed to its relatively high volatility (Vp = 14 mPa), which makes it to be mainly present in vapor phase. Azoxystrobin, which is predominantly found in the particulate phase (Vp = 1.10 × 10^−7^) showed concentrations ranging from 9.71 to 184 pg m^−3^. This fungicide has been widely used in fruit crops in the Southern region of Bahia. The concentration ranges for some pesticides determined in this work were comparable to the concentrations determined in recent studies that employed different analytical techniques (Table 5) for determination of pesticides in PM10 and PM2.5 samples.

**Table 5:** Comparison of pesticide concentrations found in this work with recent studies.

Reference	Pesticides					
	Azoxystrobin	Bifenthrin	Carbofuran	Chloropyrifos	Demeton-O	Diazinon
	Average/Range (pg m^−3^)					
This Work (PM2.5)	59.2/9.71–184	39.2/14.5–72.5	20.0/12.3–37.1	35.4/23.8–47.1	28.7/16.1–55.5	nd − < LQ
Coscollà *et al*.^47^ (PM2.5)	8.3/8.2–8.4	—	—	—	—	—
Lopez *et al*.^31^ (PM10)	160/ > LQ-800	18/ < LQ-80	18/ < LQ-46	70/LQ-210	—	20/ < LQ-220
Yusà *et al*.^30^ (PM10)	—	25.53/1.92–83.38	7.38/3.00–15.54	17.9/1.33–625.8	—	30.31/8.07–176.1
Coscollà *et al*.^38^ (PM10)	7.4/6.9–8.3	—	13/6.8 – 460	—	—	—
Borrás *et al*.^49^ (PM10)	—	—	—	NA/220-2660	—	—
Coscollà *et al*.^37^ (PM10)	1210/660–1790	—	—	2860/110–97770	—	890/280–1490
Coscollà *et al*.^29^ (PM10)	—	24.59/1.64–83.38	—	122.1/1.33–625.8	—	58.35/8.07–252.94
Hart *et al*.^19^ (PM10)	—	17.7/1.9–83.4	—	14.5/1.3–210.6	—	20.8/3.7–216.6
**Reference**	**Pesticides (continued)**					
	**Disulfoton**	**Ethion**	**Fenthion**	**Malathion**	**Parathion**	**Permethrin**
	**Average/Range (pg m** ^**−3**^ **)**					
This Work (PM2.5)	nd − < LQ	35.3/9.20–94.1	nd − < LQ	27.9/13.0–66.7	55.6/18.1–95.8	315/62.1–945^a^
Coscollà *et al*.^47^ (PM2.5)	—	—	—	11.5/6.8–17.4	—	—
Lopez *et al*.^31^ (PM10)	—	—	—	12/ < LQ–90	—	—
Yusà *et al*.^30^ (PM10)	—	—	—	15.58/2.67–94.57	—	—
Coscollà *et al*.^38^ (PM10)	—	—	—	—	—	—
Borrás *et al*.^49^ (PM10)	—	—	NA/80–4460	NA/450–4500	—	—
Coscollà *et al*.^37^ (PM10)	—	—	—	210/NA	—	—
Coscollà *et al*.^29^ (PM10)	—	—	—	12.13/2.89–94.58	—	—
Hart *et al*.^19^ (PM10)	—	—	—	13.8/2.7–94.6	—	38.3/14.4 72.7

^a^Sum of the permethrin I and permethrin II isomers. NA: Data not available. nd: not detected.

### Risk assessment considering pesticides in PM2.5

The risk and exposure assessment for adults, children and infants, considering the mean and maximum concentrations of each pesticide are shown in Table 6.

**Table 6:** Maximum and average concentration (pg m^−3^) of pesticides in PM2.5 samples and values of Daily Inhalation Exposure (DIE) (mg kg^−1^ day^−1^) and Hazard Quotient (HQAOEL).

Pesticides	Level	Adults ( > 12)			Children (1–6)		Infants ( > 6–15)	
		Concentration (pg m^−3^)	DIE	HQ (AOEL)	DIE	HQ (AOEL)	DIE	HQ(AOEL)
Azoxystrobin	Maximum	184	5.26 × 10^−8^	2.63 × 10^−7^	1.23 × 10^−7^	6.13 × 10^−7^	1.5 × 10^−7^	7.36 × 10^−7^
	Mean	59.2	1.69 × 10^−8^	8.46 × 10^−8^	3.95 × 10^−8^	1.97 × 10^−7^	4.7 × 10^−8^	2.37 × 10^−7^
Bifenthrin	Maximum	72.5	2.07 × 10^−8^	2.76 × 10^−6^	4.83 × 10^−8^	6.44 × 10^−6^	5.8 × 10^−8^	7.73 × 10^−6^
	Mean	39.2	1.12 × 10^−8^	1.49 × 10^−6^	2.61 × 10^−8^	3.48 × 10^−6^	3.1 × 10^−8^	4.18 × 10^−6^
Carbofuran	Maximum	37.1	1.06 × 10^−8^	3.53 × 10^−5^	2.47 × 10^−8^	8.24 × 10^−5^	3.0 × 10^−8^	9.89 × 10^−5^
	Mean	20	5.71 × 10^−9^	1.90 × 10^−5^	1.33 × 10^−8^	4.44 × 10^−5^	1.6 × 10^−8^	5.33 × 10^−5^
Chloropyrifos	Maximum	47.1	1.35 × 10^−8^	1.35 × 10^−6^	3.14 × 10^−8^	3.14 × 10^−6^	3.8 × 10^−8^	3.77 × 10^−6^
	Mean	35.4	1.01 × 10^−8^	1.01 × 10^−6^	2.36 × 10^−8^	2.36 × 10^−6^	2.8 × 10^−8^	2.83 × 10^−6^
Demeton-O	Maximum	55.5	1.59 × 10^−8^	NA	3.70 × 10^−8^	NA	4.4 × 10^−8^	NA
	Mean	28.7	8.20 × 10^−9^	NA	1.91 × 10^−8^	NA	2.3 × 10^−8^	NA
Ethion	Maximum	94.1	2.69 × 10^−8^	NA	6.27 × 10^−8^	NA	7.5 × 10^−8^	NA
	Mean	35.3	1.01 × 10^−8^	NA	2.35 × 10^−8^	NA	2.8 × 10^−8^	NA
Malathion	Maximum	66.1	1.89 × 10^−8^	6.30 × 10^−7^	4.41 × 10^−8^	1.47 × 10^−6^	5.3 × 10^−8^	1.80 × 10^−6^
	Mean	27.9	7.97 × 10^−9^	2.66 × 10^−7^	1.86 × 10^−8^	6.20 × 10^−7^	2.2 × 10^−8^	7.40 × 10^−7^
Parathion	Maximum	95.8	2.74 × 10^−8^	NA	6.39 × 10^−8^	NA	7.7 × 10^−8^	NA
	Mean	55.6	1.59 × 10^−8^	NA	3.71 × 10^−8^	NA	4.4 × 10^−8^	NA
Permethrin	Maximum	945	2.70 × 10^−7^	2.70 × 10^−4^	6.30 × 10^−7^	6.30 × 10^−4^	7.6 × 10^−7^	7.60 × 10^−4^
	Mean	315	9.00 × 10^−8^	9.00 × 10^−5^	2.10 × 10^−7^	2.10 × 10^−4^	2.5 × 10^−7^	2.50 × 10^−4^

NA: AOEL values no available for theses pesticides.

Considering the maximum exposure scenario (pesticides at the maximum level), the DIE ranged from 5.26 × 10^−8^ (azoxystrobin) to 2.70 × 10^−7^ mg kg^−1^ day^−1^ (permethrin) for adults, from 6.27 × 10^−8^ (ethion) to 1.23 10^−7^ mg kg^−1^ day^−1^ (azoxystrobin) for children and 7.66 × 10^−8^ (permethrin) to 1.47 × 10^−7^ mg kg^−1^ day^−1^ (azoxystrobin) for infants. Considering that HQ > 1 indicates a potential risk, the estimated HQ (AOEL) was always lower than 2.70 × 10^−4^, 2.10 × 10^−4^ and 2.50 × 10^−4^ for adults, children and infants, respectively. This means there is not a potential risk for those subjects for the sampling period considered in this study. These values were lower than those determined by Yusà *et al*.^30^, which HQ ranging from 1.40 × 10^−4^ to 1.90 × 10^−4^ was found for adults, children and infants, respectively. However, the values found in this study were similar to those determined by Lopéz *et al*.^31^ who estimated HQ for adults, children and infants in rural and remote regions and found values ranging from 4.10 × 10^−4^ to 5.03 × 10^−4^. For pesticides with common action mode cumulative exposure was calculated using a hazard index (HI). The cumulative exposure (maximum concentration scenario), adding the contributions of organophosphate pesticides (carbofuran, chloropyrifos and malathion) was 3.73 × 10^−5^, 8.71 × 10^−5^ e 1.04 × 10^−4^ for adults, children and infants, respectively. Higher values were found for pyrethroid pesticides (permethrin and bifenthrin), where HI was 2.73 × 10^−4^ for adults, 6.36 × 10^−4^ children and 7.64 × 10^−4^ for infants. Considering there is a potential hazard when HI > 1, the cumulative risk exposure for these compounds is within acceptable range.

For the potentially carcinogenic pesticides bifenthrin, malathion, parathion and permethrin were calculated the risks of cancer for children and infants, who are most vulnerable individuals. The calculated values ranged from 6.39 × 10^−4^ (parathion) to 7.60 10^−8^ (permethrin). Although permethrin is considered likely to be carcinogenic to humans^51^, the concern about the cancer risk occurs when the estimated value reaches^30^ 1 × 10^−6^. Therefore, the values found in this work were lower than the established maximum limit.

## Concluding remarks

A new, simple and miniaturized method of ultrasonic assisted extraction was developed, validated and successfully applied for determination of 13 pesticides of different chemical classes in fine particulate matter (PM2.5). The method presented high efficiency, being possible to obtain high analytical frequency in comparison to other methodologies available in the literature for the analysis of pesticides. The proposed procedure also presented minimum consumption of solvent, energy and time (high analytical frequency), and the ease for miniaturization and automation, according to the principles of Green Chemistry.

The procedure was applied in the determination of organophosphate pesticides, pyrethroids, carbamates and estrubirulins in 41 samples collected at three different sites of Todos os Santos Bay. The concentrations obtained were comparable to other studies recently published elsewhere, with different analytical techniques. As far as we know, this is the first time PM2.5 pesticides were characterized in the Southern Hemisphere, specifically in a tropical coastal area. Since we studied pesticides in PM2.5, we could estimate some health endpoints concerns through inhalation. Certainly, this study is representative for future studies.

A risk assessment was performed for exposure of adults, children and infants living around Todos Santos Bay region to pesticides in PM2.5. Estimates calculated for Hazard Quotient (HQ) and Risk Indexes (HI) for cumulative exposure were lower than 1, showing that there is no significant concern regarding exposure risk in the Todos os Santos Bay considering the daily inhalation. Cancer risks for possibly carcinogenic pesticides were calculated and values were below 6.40 × 10^−9^ for children and infants.

## Methods

### Reagents and standards

Malathion, parathion, sulfotep, fenthion, disulfoton, demeton-O, chloropyrifos and bifenthrin certified analytical standards were purchased from AccuStandard (New Haven, CT, USA). In addition, ethion, carbofuran and azoxystrobin standards were acquired from Sigma-Aldrich (St. Louis, USA) and permethrin acquired from Supelco (St. Louis, USA). All standards presented purity grade of 97% or higher. Acetonitrile (99.99%) (JT Baker, USA), dichloromethane (99.9%) (JT Baker, USA) and methanol (99.9%) (Merck, Darmstadt, Germany) used in this study were spectroscopic and chromatographic grades.

Stock solutions were prepared in methanol at 500 mg L^−1^ for carbofuran, diazinon, chloropyrifos, ethion, bifenthrin, permethrin and azoxystrobin. The other stock solutions were at 960 mg L^−1^ (demeton-O), 984 mg L^−1^ (fenthion), 985 mg L^−1^ (sulfotep), 990 mg L^−1^ (molinate), 998 mg L^−1^ (parathion) and 1000 mg L^−1^ for malathion. From the stock solutions, a mix working solution (1000 µg L^−1^) containing the 13 pesticides was prepared by dilution in methanol. On the other hand, matrix-matching calibration curves composed of seven concentration levels (0.5, 1.5, 2.5, 3.5, 4.5, 5.5, 6.5, and 7.5 µg L^−1^) were prepared for each compound adding variable volumes of mix working solutions in extracts of PM2.5 blank filters.

### Collection of fine particulate matter samples

A PM_2.5_ high-volume (Hi-Vol) sampler (Energética, Brazil) was utilized for collection of fine particles. Samples were collected on quartz fiber filters (20.3 cm × 25.4 cm, CAT No. 1851-865, Whatmann, USA) during 24 h, at 1.16 m^3^ min^−1^, which corresponded to total sampled air volume of 1670 m^3^.

A total of 41 PM2.5 samples were collected from July to October 2010 in three different sites (Maré Island, Navy Base of Aratu and Itaparica Island) located around of Todos os Santos Bay region (TSB), in the State of Bahia, Northeastern Brazil. The TSB is located around Salvador city and its metropolitan area, which is the main city of the State of Bahia, and it is situated between latitude South 12° 50′ and longitude West 38° 38′ (Headquarter of the Amazon Blue). TSB is the second largest Brazilian Bay, possessing 1,233 km² of total area. There are approximately three million inhabitants living in its surroundings.

After sampling, filters were folded in half, face-to-face, and placed in an aluminum foil envelop and then in a zip lock type plastic bag. Samples were transported cool to the laboratory and stored in a freezer at −4 °C until analysis.

The mass of the filters before and after aerosol collection was determined gravimetrically using an electronic analytical balance with a sensitivity of 0.1 mg (Sartorius, Germany). Before each weighing, filters were conditioned for 24 h in a chamber with a relative humidity of 40% and a temperature of 25 °C.

### Miniaturized ultrasound-assisted extraction

The extraction of PM_2.5_ samples were performed using a syringeless microextraction device (Whatmann Mini Uniprep G2 Filters, Maidstone, UK) with objective of miniaturizing the extraction procedure. Firstly, we tested two devices namely: a polypropylene chamber microextractor; and a glass chamber microextractor, in regard to add possible interferents to the extracts during sample preparation. The device consisted of two parts, a high density polypropylene chamber with 0.5 mL capacity and a polypropylene plunger consisting of a polyvinylidene difluoride (PVDF) filtration membrane (0.22 µm pore size) and a lid with a pre-attached silicone septum. In turn, the glass chamber device also consisted of two parts, a borosilicate glass chamber and a polypropylene plunger consisting of a polyvinylidene difluoride (PVDF) filtration membrane (0.22 µm pore size) and a lid with a pre-attached silicone septum. Both devices have the same dimensions and also follow the same extraction procedure (Supplementary Figure 2). For choosing which device would give us the best results, we monitored blank filters extracts by a GC-MS, after following the sample extraction procedure. Hereafter, we used the glass chamber device, which showed the best results (*e.g*. the least amount of interferents peaks).

For extraction procedure, a 4.15 cm^2^ filter section was cut into smaller parts and transferred into the device chamber. It was added 500 µL of a binary solvent mixture composed of 18% acetonitrile (ACN) in dichloromethane (DCM) onto sample pieces^33^ followed by closing it with the micro-extractor plunger, which was used as a lid. The whole micro-extractor systems were sonicated during 23 min using a SX-10 sonicator bath (Arruda Ultrasson, Brazil) at 26 °C and 40 kHz of potency. After that, the plunger was carefully pressed down into the glass chamber filtering the extract instantly. It was not necessary any further step for preconcentration or clean up prior analysis. Taking into account that the micro-extractor assembled-together-parts assumed the same size of a regular 2 mL vial, it was placed directly in the GC autosampler for analysis.

### Instrumentation and chromatographic analysis

In this study we adopted a modified version of the chromatographic method from Anjos & de Andrade^52, 53^. A gas chromatograph coupled to a mass spectrometer GC-MS QP2010Ultra (Shimadzu, Japan), equipped with an AOC-20i autosampler and split/splitless injector operating in splitless mode at 300 °C and purge time of 0.75 min were employed for pesticide analysis. The injection volume was 1 µL. The chromatographic separation was performed using a Rtx-5MS gas capillary column (5% diphenyl, 95% dimethylpolysiloxane, 30 m × 0.250 mm ID × 0.25 µm of film thickness) (Restek, Bellefonte, USA). High purity helium (99.9999%) (White Martins, Brazil) was used as carrier gas under flow rate of 1.00 mL min^−1^.

Oven temperature program was started in 60 °C, held it for 1.0 min, and then it was increased to 200 °C at 25 °C min^−1^, following to 280 °C at 10 °C min^−1^, and to 300 °C at 5 °C min^−1^, and finally being held at 300 °C for 1.40 min. The mass spectrometer was operated in electron impact ionization mode (IE) at 70 eV. The transfer line temperature and ion source were held at 300 °C. The total runtime was 20 min.

The separation and pesticides retention times were optimized through the scan mode with mass range between *m/z* 40 and 400 a.m.u., by injecting a 10 mg L^−1^ pesticide mix analytical standard. After this step, in order approach unequivocal peak identification, the SIM (Selected Ion Monitoring) mode was utilized and three specific ions were chosen for each analyte (one base ion and two reference ions). However, for quantification we used the peak area from the base ion only. For permethrin, which presents stereoisomerism, two peaks were detected, corresponding to the *cis (Z)* and *trans (E)* isomers. The parameters of GC-MS analysis are shown in Table 1.

### Quality control protocol and method validation

All analytical signals from samples have been corrected taking into account the instrument, reagent and method blanks. The instrument blank (carrier gas of GC-MS) was analyzed in order to evaluate the contamination of the system in the absence of any sample. The reagent or solvent blank was evaluated by analyzing only the reagents used in the extraction procedure. The method blank was evaluated by the extraction of a blank filter (filter without particles), extracted under the same conditions of a real sample. We also checked out the field blanks. If any signal was detected in any of these blanks, we discounted them from the signals found in real samples.

The method was validated taking in account the following figures-of-the-merit: linear range, linearity, limit of detection (LOD), limit of quantification (LOQ), precision (expressed as intraday and interday precisions) and accuracy (measured as mean recovery levels).

Linearity was evaluated within an analytical curve (in duplicate) composed of seven levels of concentrations for each compound. A lack of fit test was applied for each calibration curves as recommended by IUPAC^54^. In turn, intraday precision was done by the relative standard deviation (RSD) of nine injections of 3.5 µg L^−1^ mix-working solution of pesticides within a day. In turn, the interday precision was calculated by RSD from the nine injections 3.5 µg L^−1^ mix-working solution in three consecutive days (27 injections in total). The LOD and LOQ were obtained based on the parameters of the calibration curve, as described by Ribani *et al*.^55^. In this study, LOD and LOQ were calculated by the following equations: LOD = 3 × (SB/a) and LOQ = 10 × (SB/a), where “SB” refers to the standard deviation of the linear coefficient and “a” was the slope of the calibration curve.

Due to the absence of certified reference material of particulate matter for pesticides, accuracy was evaluated by recovery tests by two ways: firstly, we did the recovery tests by adding a known amount of each pesticide onto blank filters, with final concentrations of 1.5, 3.5 and 6.5 µg L^−1^ for each substance, with two replicates in each concentration level. Then, by addition of a known amount of each pesticide onto 1 mg urban dust SRM 1649b, with final concentration of 18 µg L^−1^. Finally, we applied our method for determination of pesticides in real PM_2.5_ samples.

### Risk assessment and chronic exposure to pesticides in PM2.5

In order to evaluate the risks related to the exposure of the inhabitants living around the Todos os Santos Bay to the pesticides found in PM2.5 samples, we performed a risk and chronic exposure assessment^30, 31^ associated to the fine particulate material (PM2.5). Inhalation is the main route of exposure of peoples to pesticides in the air. To estimate the inhalation exposure from pesticides in PM2.5, the following equation was considered^30, 31, 51, 56^.

1 $${\rm{DIE}}\,({\rm{mg}}\,{{\rm{kg}}}^{-1}{{\rm{day}}}^{-1})=({{\rm{C}}}_{({\rm{PM}}2.5)}\times {{\rm{IR}}}_{{\rm{inh}}}\times {\rm{ED}})/{\rm{BW}}$$

where DIE is the daily inhalation exposition, C_(PM2.5)_ is the concentration of each pesticide in PM_2.5_ samples (mg m^−3^), IR_inh_ is the inhalation rate per hour (m^3^ h^−1^), ED is exposure duration to air (h) and BW is the body weight of a subject (kg).

The risk assessment was performed considering three different groups of people: adults (>12 years), children (1–6 years) and infants (6 months–1.5 years) inserted in two different exposure scenarios: A) using the maximum concentration of each pesticide found during the sampling period and b) using the mean concentrations found in this study. In both scenarios, ED = 24 h and exposure frequency of 1 (equivalent to 12 months per year) were considered^30^. The IR_inh_ for adults, children and infants was 20, 10 and 8 m^3^ day^−1^, respectively. The BW was considered 70 kg for adults, 15 kg for children and 10 kg for infants^56^. The hazard quotient (HQ) was utilized as a risk descriptor (applied to populations living in the surroundings of Todos os Santos Bay), being calculated according to Equation 2:

2 $${\rm{HQ}}={{\rm{DIE}}}_{{\rm{i}}}/{{\rm{HBRV}}}_{{\rm{i}}}$$

where HBRV_i_ is Health Based Reference Values (as mean Acceptable Operator Exposure Level - AOEL), which were obtained from European Union Pesticides Database^20^ and the International Union of Pure and Applied Chemistry Pesticides Database^9^.

When HQ >1 a potential health risk should be considered. For pesticides with common action mode such as organophosphates, pyrethroids and carbamates, it is possible a cumulative effect. In these cases, the hazard index (HI) is calculated using Equation 3:

3 $${\rm{HI}}={\rm{HQ}}1\,({\rm{pesticide}}\,1)+{\rm{HQ}}2\,({\rm{pesticide}}\,2)+({\rm{pesticide}}\,3)+\ldots \,({\rm{pesticide}}\,{\rm{n}})$$

For pesticides considered to be carcinogenic according to the classification of the Annual Cancer Report^51^ the risk of cancer was estimated according to Equation 4:

4 $${\rm{Cancer}}\,{\rm{risk}}={\rm{DIE}}\,{(\mathrm{mg\; kg}}^{-1}{{\rm{day}}}^{-1})\times {\rm{PF}}\,{(\mathrm{mg\; kg}}^{-1}{{\rm{day}}}^{-1})$$

where PF is the Potency Factor. According *Gunier et al*.^57^ and previous works^30, 31^ PF for possibly carcinogenic pesticides ranges from >0.01 to 0.1, therefore, the value 0.1 was utilized for bifenthrin, malathion, parathion and permethrin.

## Electronic supplementary material

Supplementary Material
