# Supplementary material for: Pesticides in fine airborne particles: from a green analysis method to atmospheric characterization and risk assessment
Source: Sci Rep. 2017 May 23;7:2267. doi: 10.1038/s41598-017-02518-1 (PMC5442112; doi:10.1038/s41598-017-02518-1)
Supplement: Supplementary file 1 — Supplementary Material [file 41598_2017_2518_MOESM1_ESM.doc]

**Supplementary Information**

# Pesticides in fine airborne particles: from a green analysis method to atmospheric characterization and risk assessment

# Madson M. Nascimento^1,2,3^,, Gisele O. da Rocha^1,2,3^, Jailson B. de Andrade^1,2,3^.

_1_Instituto de Química, Universidade Federal da Bahia, Campus de Ondina, 40170-115, Salvador-BA, Brazil.

^2^Instituto Nacional de Ciência e Tecnologia em Energia e Ambiente - INCT, Universidade Federal da Bahia, 40170-115 Salvador, BA, Brazil.

^3^Centro Interdisciplinar em Energia e Ambiente - CIEnAm, Universidade Federal da Bahia, 40170-115 Salvador, BA, Brazil.

*Corresponding author: J. B. de Andrade; E-mail: [jailsondeandrade@gmail.com](mailto:jailsondeandrade@gmail.com); Fax: +55 71 3283 6821.

**A**

**B**

**Figure S1**. Chromatograms of obtained in SIM mode using two different types of microextraction devices. **A** – Chromatogram of a blank sample extraction using polypropylene device. **B** – Extraction of a blank sample using borosilicate glass device.


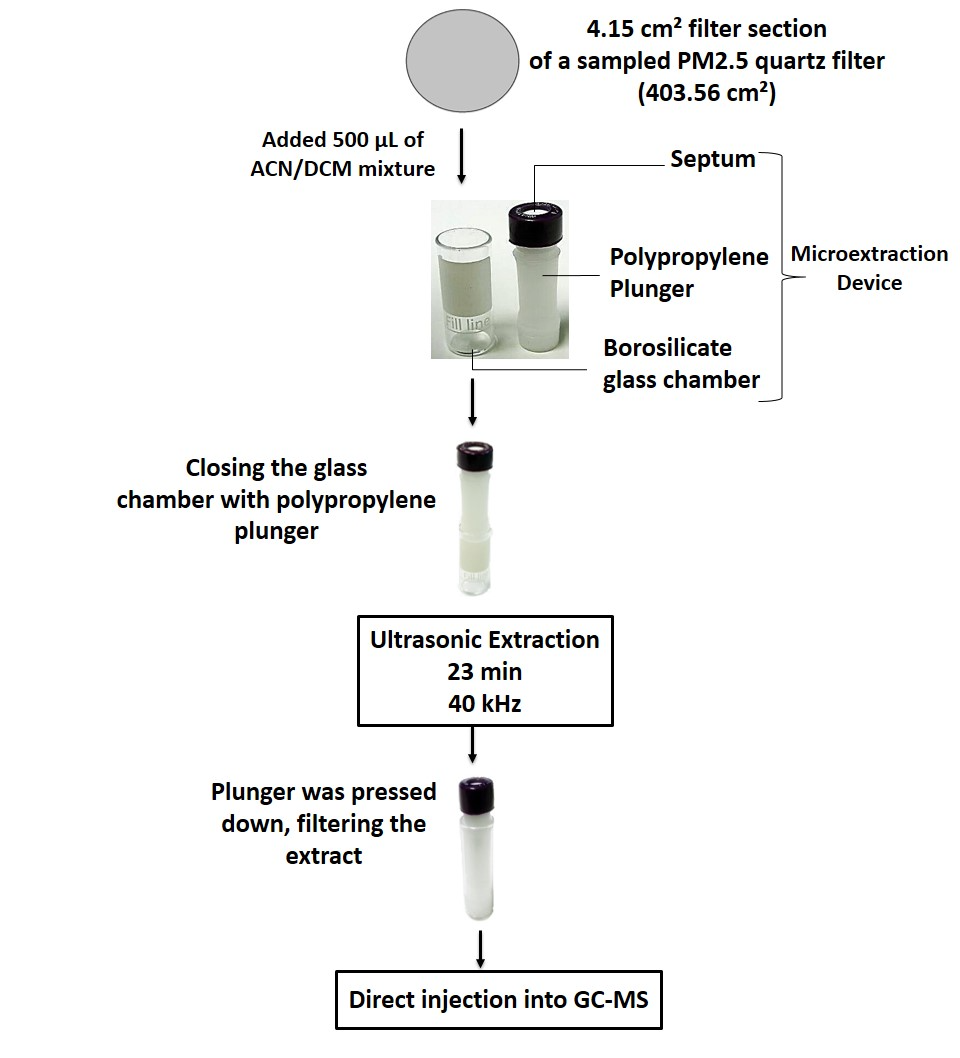


**Figure S2**. Scheme showing PM2.5 sample extraction using the microextraction device. Comparison between two different types of microextraction devices. Microextraction device, consisting of a silicone septum, a polypropylene plunger constituted of a polyvinylidene diflouride (PVDF) filtration membrane with 0.2 µm pore size and a borosilicate glass chamber (0.5 mL capacity).

| **Pesticide** | **Molecular** | **Molecular Mass** | **Chemical** | **Pesticide type** | **Vapour** | **Henry's Law** | **Water** |
| --- | --- | --- | --- | --- | --- | --- | --- |
|  | **Formula** | **(g mol^-1^)** | **Groups** |  | **Pressure** | **Constant** | **Solubility** |
|  |  |  |  |  | **(mPa)** | **(Pa m^3^ mol^-1^)** | **(mg L^-1^) at 20°C** |
| Azoxystrobin | C_22_H_17_N_3_O_5_ | 403.40 | Strobilurin | Fungicide | 1.10 x 10^-7^ | 7.40 x 10^-9^ | 6.7 |
| Bifenthrin | C_23_H_22_ClF_3_O_2_ | 422.88 | Pyrethroid | Inseticide, Acaricide | 1.78 x 10^-2^ | 7.74 x 10^-5^ | 0.001 |
| Carbofuran | C_12_H_15_NO_3_ | 221.26 | Carbamate | Inseticide, Acaricide | 8.00 x 10^-2^ | 5.0 x 10^-5^ | 322 |
| Chloropyrifos | C_9_H_11_Cl_3_NO_3_PS | 350.59 | Organophosphate | Inseticide, Acaricide | 1.43 | 0.478 | 1.05 |
| Demeton-O | C_6_H_15_O_3_PS_2_ | 230.29 | Organophosphate | Inseticide, Acaricide | not informed | not informed | 330 |
| Diazinon | C_12_H_21_N_2_O_3_PS | 304.35 | Organophosphate | Inseticide, Acaricide | 11.97 | 6,09 x10^-2^ | 60 |
| Disulfoton | C_8_H_19_O_2_PS_3_ | 274.40 | Organophosphate | Inseticide, Fungicide | 7.20 | 0.160 | 25 |
| Ethion | C_9_H_22_O_4_P_2_S_4_ | 384.48 | Organophosphate | Inseticide, Acaricide | 0.2 | 3.85 x 10^-2^ | 2.00 |
| Fenthion | C_10_H_15_O_3_PS_2_ | 278.33 | Organophosphate | Inseticide, Acaricide | 0.37 | 2.40 x 10^-2^ | 4.2 |
| Malathion | C_10_H_19_O_6_PS_2_ | 330.36 | Organophosphate | Inseticide, Acaricide | 3.1 | 1.00 x 10^-3^ | 148 |
| Parathion | C_10_H_14_NO_5_PS | 291.27 | Organophosphate | Inseticide, Acaricide | 0.89 | 3.02 x 10^-2^ | 12.4 |
| Permethrin | C_21_H_2_OCl_2_O_3_ | 391.30 | Pyrethroid | Inseticide, Acaricide | 7.0 x 10^-3^ | 1.89 x 10^-1^ | 0.2 |
| Sulfotep | C_8_H_20_O_5_P_2_S_2_ | 322.32 | Organophosphate | Inseticide | 14 | 4.50 x 10^-1^ | 10 |

**Table S1**. Physicochemical properties of the analyzed pesticides^1^.

**Reference**

1. International Union of Pure and Applied Chemistry (IUPAC). Global availability of information on agrochemicals http://sitem.herts.ac.uk/aeru/iupac/atoz.htm (2017). (Accessed 14.01.2017)
